# Clinical practice guideline recommendations for diagnosis and management of anxiety and depression in hospitalized adults with delirium: a systematic review

**DOI:** 10.1186/s13643-023-02339-6

**Published:** 2023-09-25

**Authors:** Therese G. Poulin, Natalia Jaworska, Henry T. Stelfox, Kirsten M. Fiest, Stephana J. Moss

**Affiliations:** 1 Department of Community Health Sciences, Cumming School of Medicine, University of Calgary, Calgary, AB Canada; 2 Department of Critical Care Medicine, Cumming School of Medicine, University of Calgary, Calgary, AB Canada; 3 Alberta Health Services, Calgary, AB Canada; 4 O’Brien Institute for Public Health, University of Calgary, Calgary, AB Canada; 5 Department of Psychiatry, Cumming School of Medicine, University of Calgary, Calgary, AB Canada; 6 Hotchkiss Brain Institute, University of Calgary, Calgary, AB Canada

**Keywords:** Delirium, Clinical practice guideline, Anxiety, Depression, Systematic review, Mental health

## Abstract

**Background:**

Delirium commonly occurs in hospitalized adults. Psychiatric disorders such as anxiety, depression, and post-traumatic stress disorder (PTSD) can co-occur with delirium, and can be recognized and managed by clinicians using recommendations found in methodological guiding statements called Clinical Practice Guidelines (CPGs). The specific aims of this review were to: [1] synthesize CPG recommendations for the diagnosis and management of anxiety, depression, and PTSD in adults with delirium in acute care; and [2] identify recent published literature in addition to those identified and reported in a 2017 review on delirium CPG recommendations and quality.

**Methods:**

MEDLINE, EMBASE, CINAHL, PsycINFO, and 21 sites on the Canadian Agency for Drugs and Technologies listed in the Health Grey Matters Lite tool were searched from inception to February 12, 2021. Selected CPGs focused on delirium in acute care, were endorsed by an international scientific society or governmental organization, and contained at least one recommendation for the diagnosis or management of delirium. Two reviewers independently extracted data in duplicate and independently assessed CPG quality using the AGREE-II tool. Narrative synthesis of CPG recommendations was conducted.

**Results:**

Title and abstract screening was completed on 7611 records. Full-text review was performed on 197 CPGs. The final review included 27 CPGs of which 7 (26%) provided recommendations for anxiety (4/7, 57%), depression (5/7, 71%), and PTSD (1/7, 14%) in delirium. Twenty CPGs provided recommendations for delirium only (e.g., assess patient regularly, avoid use of benzodiazepines). Recommendations for the diagnosis of psychiatric disorders with delirium included using evidence-based diagnostic criteria and standardized screening tools. Recommendations for the management of psychiatric disorders with delirium included pharmacological (e.g., anxiolytics, antidepressants) and non-pharmacological interventions (e.g., promoting patient orientation using clocks). Guideline quality varied: the lowest was Applicability (mean = 36%); the highest Clarity of Presentation (mean = 76%).

**Conclusions:**

There are few available evidence-based CPGs to facilitate appropriate diagnosis and management of anxiety, depression, and PTSD in patients with delirium in acute care. Future guideline developers should incorporate evidence-based recommendations on the diagnosis and management of these psychiatric disorders in delirium.

**Systematic review registration:**

Registration number: PROSPERO (CRD42021237056)

**Supplementary Information:**

The online version contains supplementary material available at 10.1186/s13643-023-02339-6.

## Background

Delirium is an acute and complex neuropsychiatric disorder that commonly affects hospitalized patients [1]. Delirium is the most common neuropsychiatric disorder in acute care, with prevalence estimates of 10–30% within the hospitalized population [2, 3]. Patients with delirium are often hospitalized for longer and have a poorer survival prognosis than those without delirium, and the risk of developing delirium generally increases with the severity of illness [4]. Identifying and implementing effective strategies to mitigate the risk of delirium are essential to reducing long-term morbidity related to critical illness [5].

Delirium and other neuropsychiatric disorders are often similar in presentation, often present concurrently, and patients may have more than one type of disorder [6]. There is difficulty distinguishing these disorders: for example, severe hypoactive delirium [7] can be confused with depression, and hyperactive delirium [8] can be confused with mania. Patients should be evaluated for delirium and psychiatric disorders to not to miss important medical problems; a lack of recognition for pre-existing and new psychiatric disorders during an acute care admission may contribute to poor patient mental health and increased severity of psychiatric disorder symptoms [9–11].

A Clinical Practice Guideline (CPG) is a methodological statement aimed at providing guidance to clinicians and their patients for specific medical circumstances and conditions [12]. CPG use can support the reduction of financial cost from inappropriate care and can improve clinical decision-making and quality of care for clinicians and patients [13]. A recent systematic review of the quality of CPGs for delirium in acute care evaluated the quality of guideline recommendations focusing on knowledge translation resources and the practical application and monitoring of guideline implementation [14]. The most recent included CPG in this review was published in 2013 thereby necessitating an updated review to identify progress and/or gaps in our evidence base on this topic.

The first objective of our systematic review is to add an analysis, synthesis, and quality assessment of the available CPGs on recommendations for the identification and management of anxiety, depression, and PTSD in adults with delirium in acute care. The second objective is to provide an updated synthesis and quality assessment of CPG recommendations for delirium identification and management in acute care.

## Methods

This systematic review was registered on PROSPERO (CRD42021237056) prior to data abstraction and reported using the Preferred Reporting Items for Systematic Reviews and Meta-Analyses (PRISMA) guidelines [15] (Additional file 1: Appendix 1).

### Identification and selection of studies

We performed systematic searches of MEDLINE, EMBASE, PsycINFO and CINAHL from inception to February 12, 2021, to identify eligible CPGs. We additionally performed a comprehensive search of the grey literature using the 21 sites listed on the Canadian Agency for Drugs and Technologies in Health (CADTH) Grey Matters Lite tool [16] from inception to February 12, 2021. Search strategies for each database are included in Additional file 2: Appendix 2. A librarian (N.D.) performed an independent PRESS review [17] of the EMBASE search strategy. CPG reference lists were screened for additional guidelines relevant to the review which may have been missed by the search. No limits (e.g., date, language) were applied to any search.

### Study eligibility

Two reviewers (T.G.P., S.J.M.) assessed record title and abstract eligibility independently and in duplicate. Guidelines eligible for inclusion were written in English, were issued or endorsed by a national or international scientific society or government organization and had a primary focus on the diagnosis and/or management of delirium in any acute care setting. Guidelines eligible for inclusion contained at least one recommendation on the diagnosis, prevention, or management of delirium presented within guideline text, tables, figures, algorithms, and/or decisions paths. Guideline recommendation(s) on delirium must: (1) be accompanied with an explicit level of confidence (i.e., the GRADE system [18]); and (2) explicitly discuss one or more interventions for the recognition or management of delirium. Comparison of these recommended interventions to other interventions was not required. Guidelines with additional recommendations pertaining to anxiety, depression, or PTSD were of special interest. Any year of publication, publishing region and guideline development process were of interest. The most updated versions of guidelines were included in the review. Title and abstracts were advanced for full-text review if both reviewers agreed independently and in duplicate that they satisfied one or more of the eligibility criteria. Full-text guidelines were included in the review if both reviewers agreed independently and in duplicate that they met all the criteria for inclusion. Discrepancies were handled through discussion with a third reviewer (K.D.K.).

### Data extraction and synthesis

Data extraction was completed independently and in duplicate by two reviewers (T.G.P. and S.J.M.). Data extracted from included guidelines consisted of guideline name, author(s), development group, country, language(s), target population(s), evidence consensus method(s) and psychiatric disorder(s). Narrative synthesis of recommendations for diagnosis and management of delirium and of psychiatric disorders was completed after data extraction for relevant guidelines. Recommendations for diagnosis and management of any symptoms of anxiety, depression, and PTSD in delirium were included, in addition to recommendations for the management of pre-existing or new diagnoses of these disorders.

### Guideline quality assessment using AGREE II

The Appraisal of Guidelines for Research and Evaluation II (AGREE II) Instrument [19] is designed to assess the methodological quality and reporting foundation of a CPG. The AGREE II tool is composed of a total of 23 items in 6 Domains and 2 overall global rating items. All items are rated on a seven-point scale (1 = no information or poorly reported, 7 = reporting quality is exceptional and meets all criteria); except for the second global rating item which asks if the rater would recommend the guideline for use (yes, yes with modification, or no). The six Domains include: Domain 1, Scope and Purpose (assesses the overall guideline goal and target population as well as the health questions); Domain 2, Stakeholder Involvement (targets the participants involved in the guideline development group and how the guideline represents the perspectives of users); Domain 3, Rigor of Development (assesses how the evidence was gathered, expressed, and how it will be updated); Domain 4, Clarity of Presentation (reviews guideline organization and language); Domain 5, Applicability (assesses if implementation is feasible, the economic consequences, and if there are specific strategies for implementation); and Domain 6, Editorial Independence (measures the level of independence from funding institutions and competing interests of guideline developers). Two reviewers (T.G.P. and S.J.M.) scored all included guidelines independently and in duplicate, and any discrepancies were resolved through discussion. As suggested by the AGREE II tool [19], six scaled Domain scores for each guideline were calculated by summing all individual scores in the Domain and scaling as a percentage of the maximum possible score for that Domain. For each guideline, the average overall score was determined by taking the mean of the first overall global rating item (“Rate the overall quality of this guideline from 1 to 7”) after both reviewers assigned it a score. For the overall assessment, reviewers used criteria from all Domains to judge if they would recommend the guideline for use using “yes”, “yes, with modification”, or “no”.

## Results

### Results of search

**Fig. 1:**
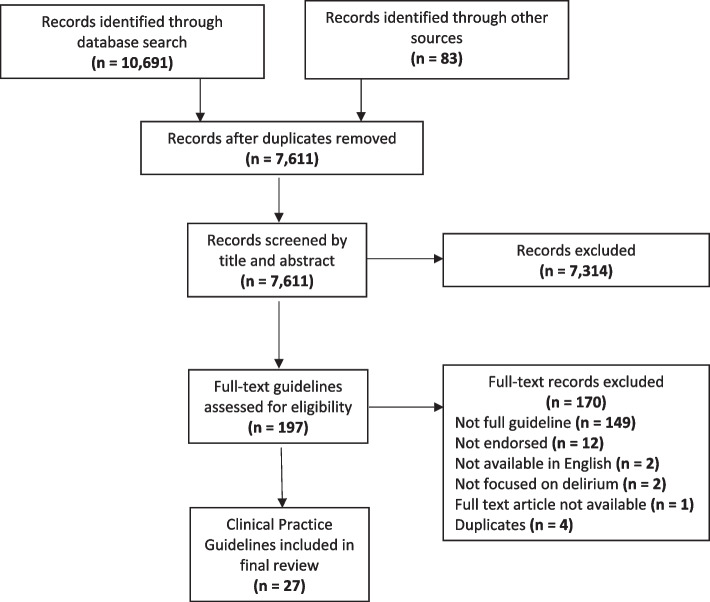
PRISMA flow diagram

Initial database search, grey literature search and reference list screening resulted in a total of 10,774 records (Fig. 1). After duplicates were removed, 7611 records were screened by title and abstract, and 7314 were excluded. Full-text review was performed on 197 records and following this, 170 records were excluded due to: not being a full guideline (e.g., not providing specific sections such as references) (*n* = 149), not being endorsed by a national or international scientific society or government organization (*n* = 12), not available in English (*n* = 2), not focused on delirium (*n* = 2), full-text unavailable (*n* = 1), and being a duplicate (*n* = 4). The final review included 27 CPGs.

### Study characteristics

Guideline characteristics are presented in Table 1. Included guidelines were published from 1999 to 2020, with two guidelines being updates of previous versions: Jacobi et al. published in 2002, updated from 1994 [20], and Devlin et al. published in 2018, updated from 2013 [21]. The most recent versions of guidelines were included in the review. Countries where guidelines were developed included Canada (*n* = 10), United States of America (*n* = 7), United Kingdom (*n* = 7), Australia (*n* = 1), Europe (*n* = 1), Germany (*n* = 1), India (*n* = 1), and Switzerland (*n* = 1). The most common guideline target populations were healthcare professionals working in general (e.g., general hospital) acute care settings (*n* = 12; 44%) and ICU healthcare professionals (*n* = 4; 15%). Informal consensus (i.e., discussion) was the most commonly used consensus method during guideline development (*n* = 12; 44%), however, this was often employed alongside formal tools such as GRADE [18], AGREE [19], or the development method and guideline classification schemes described by Shekelle et al. [22]. While all (*n* = 27) guidelines focused on delirium, 20 guidelines (74%) included recommendations on delirium only, whereas seven guideline (26%) provided additional recommendations for anxiety, depression, and PTSD in delirium.

**Table 1:** Summary of included guideline characteristics

Author	Guideline name	Development group	Year	Country	Language	Target population	Consensus method(s)	Psychiatric disorder(s)	AGREE II Rigour Domain Score
Allard et al.	Guideline on the Assessment and Treatment of Delirium in Older Adults at the End of Life	Canadian Coalition for Seniors' Mental Health	2010	Canada	English	Healthcare professionals working with older adults at the end of life with or at risk of developing delirium	Shekelle et al. method, informal consensus	Delirium	64%
American Geriatrics Society	American Geriatrics Society Abstracted Clinical Practice Guideline for Postoperative Delirium in Older Adults	American Geriatrics Society Expert Panel	2014	United Kingdom	English	Healthcare professional or healthcare system providing care for older adults in the post-surgical setting	American College of Physicians' Guideline Grading System, Cochrane Risk of Bias, Jadad scoring system	Delirium	66%
Brajtman et al.	Developing Guidelines on the Assessment and Treatment of Delirium in Older Adults at the End of Life	Guideline Adaptation Group for the Assessment and Treatment of Delirium in Older Adults at the End of Life	2011 (based on the 2006 Canadian Coalition for Seniors' Mental Health CPG)	Canada	English	NS	Informal grading scheme, informal consensus	Delirium	40%
Bush et al.	Delirium in Adult Cancer Patients: ESMO Clinical Practice Guidelines	European Society for Medical Oncology	2018	Canada, Europe	English	Healthcare professionals providing care for patients with cancer	Infectious Diseases Society of America-United States Public Health Service Grading System (adapted), ESMO faculty informal consensus	Delirium	51%
Dans et al.	NCCN Guidelines Palliative Care, Version 2.2017	National Comprehensive Cancer Network	2017	United States of America	English	Healthcare professionals providing care for patients with cancer	NS	Delirium	9%
Devlin et al.	Clinical Practice Guidelines for the Prevention and Management of Pain, Agitation/Sedation, Delirium, Immobility, and Sleep Disruption in Adult Patients in the ICU	Society of Critical Care Medicine	2018 (update from 2013)	Canada, United States of America	English	Practicing ICU clinicians	GRADE, Cochrane Risk of Bias tool, Newcastle-Ottawa scale, informal consensus using GRADE Evidence to Decision framework	Delirium	61%
ACR expert panel	ACR Appropriateness Criteria Acute Mental Status Change, Delirium, and New Onset Psychosis	American College of Radiotherapy	2019	United States of America	English	Radiologists, radiation oncologists, and referring physicians	RAND/UCLA Appropriateness Method, GRADE, expert opinion	Delirium	23%
Fraser Health	Delirium/Restlessness Symptom Guidelines	Fraser Health	2006	Canada	English	Inter-professional healthcare professionals working in various settings	NS	Delirium	23%
Grover and Avasthi	Clinical Practice Guidelines for Management of Delirium in Elderly	Department of Psychiatry, Postgraduate Institute of Medical Education & Research	2018	India	English	NS	NS	Delirium	9%
Harle et al.	Cancer Care Ontario's Symptom Management Guide-to-Practice: Delirium	Cancer Care Ontario	2010	Canada	English	Healthcare professionals providing care to patients with cancer	ADAPTE, informal consensus	Delirium	65%
Hogan and McCabe	National Guidelines for Seniors' Mental Health: The Assessment and Treatment of Delirium	Canadian Coalition for Seniors' Mental Health	2006	Canada	English	Healthcare professionals providing care to older persons	Shekelle et al. method, informal consensus, and expert opinion	Delirium	65%
Martin et al.	Evidence and Consensus-based German Guidelines for the Management of Analgesia, Sedation and Delirium in Intensive Care - Short Version	Association of Scientific Medical Societies of Germany	2010	Germany	English, German	The ICU healthcare team	Informal consensus	Delirium	29%
Michaud et al.	Delirium: Guidelines for General Hospitals	Delirium Guidelines Development Group	2007	Switzerland	English, French	Healthcare professionals	Oxford classification, RAND appropriateness method, informal consensus	Delirium	50%
Neufeld et al.	Antipsychotics for the Prevention and Treatment of Delirium	John Hopkins University Evidence-based Practice Center/Agency for Healthcare Research and Quality	2019	United States of America	English	Healthcare professionals, health system leaders and policymakers	Cochrane Risk of Bias Tool, Cochrane Risk of Bias in Non-Randomized Studies of Interventions tool	Delirium	81%
Potter et al.	The Prevention, Diagnosis and Management of Delirium in Older People: Concise Guidelines	Guideline Development Group convened by the British Geriatrics Society in conjunction with the Royal college of Physicians	2006	United Kingdom	English	Healthcare professionals and family caregivers	SIGN, AGREE, informal consensus	Delirium	30%
Thomson et al.	Diagnosis and Management of Delirium	Northern Care Alliance (NHS group)	2019	United Kingdom	English	Healthcare professionals providing care in clinical adult services	NS	Delirium	18%
Tropea et al.	Clinical Practice Guidelines for the Management of Delirium in Older People	Clinical Epidemiology and Health Service Evaluation Unit in collaboration with the Delirium Clinical Guidelines Expert Working Group	2008	Australia	English	Healthcare professionals and community care workers across Australia providing care in acute, subacute, residential and community settings	SIGN, National Health and Medical Research Council's additional levels of evidence and grades for recommendations for developers of guidelines (Pilot program 2005-2006)	Delirium	90%
Weldon et al.	Guidelines for the Prevention, Recognition and Management of Delirium in Adults in the Acute Hospital Setting	Luton and Dunstable University Hospital (NHS Foundation Trust)	2015	United Kingdom	English	Healthcare professionals providing care for patients with delirium	NS	Delirium	9%
White et al.	Guidelines for the Peri-operative Care of People with Dementia	Working Party established by the Association of Anaesthetists	2019	United Kingdom and Ireland	English	Healthcare professionals providing care in the post-surgical setting	NS	Delirium	9%
Young et al.	Delirium: Prevention, Diagnosis and Management	National Institute for Health and Care Excellence	2019	United Kingdom	English	General hospital, critical care, and long-term residential healthcare professionals and family caregivers providing care for people with or are at high risk of delirium	GRADE	Delirium	63%
Andersen et al.	Screening, Assessment, and Care of Anxiety and Depressive Symptoms in Adults with Cancer: An American Society of Clinical Oncology Guideline Adaptation	American Society of Clinical Oncology	2014	United States of America	English	Healthcare professionals and family caregivers providing care for adults with cancer	ADAPTE, informal consensus	Delirium, Depression, Anxiety	51%
BC Guidelines and Protocols Advisory Committee	Palliative Care for the Patient with Incurable Cancer or Advanced Disease Part 2: Pain and Symptom Management	BC Guidelines & Protocols Advisory Committee	2017	Canada	English	NS	NS	Delirium, Depression	4%
Jacobi et al.	Clinical Practice Guidelines for the Sustained Use of Sedatives and Analgesics in the Critically Ill Adult	Society of Critical Care Medicine and the American Society of Health-System Pharmacists	2002 (update from 1994)	United States of America	English	ICU healthcare professionals	Informal grading scheme, informal consensus	Delirium, Anxiety, Post-Traumatic Stress Disorder	50%
McNeill et al.	Delirium, Dementia, and Depression in Older Adults: Assessment and Care, Second Edition	Registered Nurses Association of Ontario	2016	Canada	English	Healthcare professionals	AMSTAR, AGREE II	Delirium, Dementia, Depression	92%
NICE rapid guideline development group	COVID-19 Rapid Guideline: Managing Symptoms (including at the end of life) in the Community	National Institute for Health and Care Excellence	2020	United Kingdom	English	Healthcare professionals	Internal accuracy check by NICE and NHS England	Delirium, Anxiety	50%
Trzepacz et al.	Practice Guideline for the Treatment of Patients with Delirium	American Psychiatric Association	1999	United States of America	English	Psychiatrists	NS	Delirium, Depression, Anxiety, Dementia	36%
Virani et al.	Caregiving Strategies for Older Adults with Delirium, Dementia and Depression	Registered Nurses Association of Ontario	2004	Canada	English	Registered nurses and registered practical nurses	AGREE, informal consensus	Delirium, Dementia, Depression	60%

*Abbreviations*: *ACR* American College of Radiology, *AGREE* Appraisal of Guidelines Research and Evaluation, *AMSTAR* Assessing the methodological quality of systematic reviews, *ASCO* American Society of Clinical Oncology, *BC* British Colombia, *COVID-19* Coronavirus disease of 2019, *CPG* Clinical practice guideline, *ESMO* European Society for Medical Oncology, *GRADE* Grading of Recommendations, Assessment, Development and Evaluation, *ICU* Intensive care unit, *NCCN* National Comprehensive Cancer Network, *NHS* National Health Service, *NICE* National Institute for Health and Care Excellence, *NS* Not specified, *PTSD* Post-traumatic stress disorder, *RAND* Research And Development, *SIGN* Scottish Intercollegiate Guidelines Network, *UCLA* University of California Los Angeles

Sorted by psychiatric disorders (delirium only guidelines listed first) then alphabetically by author

### Quality of included guidelines

Individual and cumulative guideline Domain scores assessed by the AGREE II tool are presented in Table 2. The lowest cumulative Domain was Domain 6, Editorial Independence, with a mode of 4% and a range of 4–92%. The highest cumulative Domain was Domain 4, Clarity of Presentation, with a mode of 92% and a range of 47–100%. The guideline by McNeill et al. [23] scored the highest in all Domains compared to all other guidelines: Domains 1–4 and 6 scored 92% while Domain 5 scored 71%. In addition, this guideline received an overall score of 6, and both reviewers recommended the guideline for use.

**Table 2:** AGREE II domain percentages for guidelines containing recommendations for delirium or psychiatric disorders in delirium

Author	Guideline name	Domain 1: scope and purpose (3 items)	Domain 2: stakeholder involvement (3 items)	Domain 3: rigour of development (8 items)	Domain 4: clarity of presentation (3 items)	Domain 5: applicability (4 items)	Domain 6: editorial independence (2 items)	Overall score (average)^a^	Overall recommendation for use
*Delirium Only*									
Allard et al.	Guideline on the Assessment and Treatment of Delirium in Older Adults at the End of Life	78%	53%	64%	81%	31%	38%	4.5	Yes, with modification
American Geriatrics Society	American Geriatrics Society Abstracted Clinical Practice Guideline for Postoperative Delirium in Older Adults	83%	58%	66%	83%	27%	79%	5	Yes, with modification
Brajtman et al.	Developing Guidelines on the Assessment and Treatment of Delirium in Older Adults at the End of Life	64%	42%	40%	67%	4%	67%	4	Yes, with modification
Bush et al.	Delirium in adult cancer patients: ESMO Clinical Practice Guidelines	58%	53%	51%	81%	29%	42%	4.5	Yes, with modification
Dans et al.	NCCN Guidelines Palliative Care, Version 2.2017	61%	42%	9%	47%	13%	63%	2	No
Devlin et al.	Clinical Practice Guidelines for the Prevention and Management of Pain, Agitation/Sedation, Delirium, Immobility, and Sleep Disruption in Adult Patients in the ICU	61%	81%	61%	67%	60%	75%	6.5	Yes
ACR Expert Panel	ACR Appropriateness Criteria Acute Mental Status Change, Delirium, and New Onset Psychosis	56%	47%	23%	83%	6%	42%	3.5	Yes, with modification
Fraser Health	Delirium/Restlessness Symptom Guidelines	89%	14%	23%	75%	17%	4%	4	Yes, with modification
Grover and Avasthi	Clinical Practice Guidelines for Management of Delirium in Elderly	44%	14%	9%	47%	15%	4%	2	No
Harle et al.	Cancer Care Ontario's Symptom Management Guide-to-Practice: Delirium	72%	47%	65%	81%	42%	25%	4	Yes, with modification
Hogan and McCabe	National Guidelines for Seniors' Mental Health: The Assessment and Treatment of Delirium	86%	92%	65%	86%	42%	46%	5.5	Yes, with modification
Martin et al.	Evidence and Consensus-based German Guidelines for the Management of Analgesia, Sedation and Delirium in Intensive Care - Short Version	67%	36%	29%	67%	50%	63%	3.5	Yes, with modification
Michaud et al.	Delirium: Guidelines for General Hospitals	44%	47%	50%	56%	31%	4%	5	Yes, with modification
Neufeld et al.	Antipsychotics for the Prevention and Treatment of Delirium	94%	86%	81%	92%	44%	63%	6	Yes, with modification
Potter et al.	The Prevention, Diagnosis and Management of Delirium in Older People: Concise Guidelines	56%	64%	30%	58%	33%	42%	3.5	Yes, with modification
Thomson et al.	Diagnosis and Management of Delirium	67%	42%	18%	72%	35%	4%	4	Yes, with modification
Tropea et al.	Clinical Practice Guidelines for the Management of Delirium in Older People	94%	69%	90%	92%	10%	63%	6.5	Yes, with modification
Weldon et al.	Guidelines for the Prevention, Recognition and Management of Delirium in Adults in the Acute Hospital Setting	61%	31%	9%	78%	42%	4%	3.5	No
White et al.	Guidelines for the Peri-operative Care of People with Dementia	44%	42%	9%	61%	23%	17%	2	No
Young et al.	Delirium: Prevention, Diagnosis and Management	100%	53%	63%	100%	77%	79%	6	Yes
*Anxiety, depression, or post-traumatic stress disorder in delirium*									
Andersen et al.	Screening, Assessment, and Care of Anxiety and Depressive Symptoms in Adults with Cancer: An American Society of Clinical Oncology Guideline Adaptation	94%	75%	51%	89%	46%	83%	6	Yes
BC Guidelines & Protocols Advisory Committee	Palliative Care for the Patient with Incurable Cancer or Advanced Disease Part 2: Pain and Symptom Management	83%	14%	4%	75%	52%	4%	3	Yes, with modification
Jacobi et al.	Clinical Practice Guidelines for the Sustained Use of Sedatives and Analgesics in the Critically Ill Adult	81%	47%	50%	92%	52%	4%	5.5	Yes, with modification
McNeill et al.	Delirium, Dementia, and Depression in Older Adults: Assessment and Care, Second Edition	92%	92%	92%	92%	71%	92%	6	Yes
NICE rapid guideline development group	COVID-19 Rapid Guideline: Managing Symptoms (including at the end of life) in the Community	67%	36%	50%	86%	29%	4%	4.5	Yes
Trzepacz et al.	Practice Guideline for the Treatment of Patients with Delirium	44%	31%	36%	69%	27%	29%	3.5	Yes, with modification
Virani et al.	Caregiving Strategies for Older Adults with Delirium, Dementia and Depression	89%	64%	60%	89%	56%	21%	5.5	Yes, with modification
Domain Mean		72%	51%	44%	76%	36%	39%		
Domain Mode		44%	47%	9%	92%	42%	4%		
Domain Range		44–100%	14–92%	4–92%	47–100%	4–77%	4–92%		

*Abbreviations*: *ACR* American College of Radiotherapy, *AGREE* Appraisal of Guidelines for Research and Evaluation, *BC* British Colombia, *COVID-19* Coronavirus disease of 2019, *ESMO* European Society for Medical Oncology, *ICU* Intensive care unit, *NCCN* National Comprehensive Cancer Network, *NICE* National Institute for Clinical Excellence

^a^Average scores calculated between two blinded reviewers after discussion of discrepancies

### Summary of recommendations for delirium

A synthesis of recommendations for delirium stratified by recognition and prevention is presented in Table 3. Specific sub-populations more at risk for delirium were mentioned in fewer than 60% of included guidelines: approximately half (*n* = 14; 52%) of included guidelines were designed to recognize and prevent post-operative delirium; recommendations specific to older adults (defined as > 65 years of age) were presented in 15 guidelines (56%).

**Table 3:** Synthesis of recommendations for delirium. Refer to Additional file 3: Appendix 3 for footnote details

Author	Guideline name	Recommendations for delirium	
		*Recognition*	*Prevention*
Allard et al.	Guideline on the Assessment and Treatment of Delirium in Older Adults at the End of Life	•Risk factors include socio-demographic, physical, medical, and mental status, laboratory findings, surgery, and anesthesia ^a^ •Provide educational training for healthcare professionals ^b^ •Use validated screening tools for diagnosis ^c^	•Use nonpharmacological interventions ^d^ •Monitor environmental and patient risk factors for recurrence ^e^ •Use non-opioid analgesics first, and if an opioid is needed use the minimum effective dose •Use the lowest dose of psychotropic medications •Haloperidol is the antipsychotic drug of choice, while benzodiazepines are only recommended for alcohol or sedative withdrawal delirium •There is insufficient evidence to recommend for or against psychotropic medications for hypoactive delirium •Avoid pharmacologic interventions aggravating delirium ^f^
American Geriatrics Society	American Geriatrics Society Abstracted Clinical Practice Guideline for Postoperative Delirium in Older Adults	•Provide educational training for healthcare professionals •Evaluate reversible medical causes	•Use nonpharmacological interventions •Avoid using pharmacological interventions with a high risk of aggravating delirium •Use non-opioid analgesia for prevention of post-surgical delirium •Avoid new use of cholinesterase inhibitors and benzodiazepines •Hypoactive delirium should not be treated with benzodiazepines or antipsychotics
Andersen et al.	Screening, Assessment, and Care of Anxiety and Depressive Symptoms in Adults with Cancer: An American Society of Clinical Oncology Guideline Adaptation	•Refer patients with signs of delirium to a psychiatrist, psychologist, or equivalently trained mental health professional •Use validated screening tools	•Treat reversible medical causes of delirium first •Use nonpharmacological interventions to create a safe environment and reduce risk of harm to self and others
BC Guidelines and Protocols Advisory Committee	Palliative Care for the Patient with Incurable Cancer or Advanced Disease Part 2: Pain and Symptom Management	•Assess delirium using factors like level of consciousness, hallucinations, fluctuation in mental state or confusion	•Treat reversible medical causes of delirium if consistent with goals of care •Use non-pharmacological interventions which include changing the environment, lighting, and safety protocols •Use different pharmacological interventions if delirium is hypoactive ^g^, hyperactive ^h^, or hyperactive and a risk to self and others ^i^ •Consider palliative sedation when delirium is not reversible
Brajtman et al.	Developing Guidelines on the Assessment and Treatment of Delirium in Older Adults at the End of Life	•Detect delirium using the CAM along with other validated tools ^j^ but these should only be used as a diagnostic aid •Remain vigilant for any changes in mental status, cognition, behavior, or functional ability, and investigate any new changes •Prioritize educating healthcare professionals about the care of older adults	•Ensure adequate hydration by oral fluid intake or hypodermoclysis •Use the minimum effective dose of analgesics to control pain, and consider opioid rotation •Use antipsychotics for delirium that is not a result of alcohol or benzodiazepine withdrawal •Acquire a second professional opinion before treatment of hyperactive or mixed delirium, and treat with alternative strategies (switching antipsychotics, combining two antipsychotics including one with a sedative effect, and combining a benzodiazepine with an antipsychotic) •Minimize physical restraints and use only in exceptional circumstances
Bush et al.	Delirium in adult cancer patients: ESMO Clinical Practice Guidelines	•Risk factors include direct ^k^ (cancer related) and indirect ^l^ (secondary) complications •Diagnose delirium by administering validated clinical assessments based on DSM or ICD criteria with a trained healthcare professional •Provide delirium education for family caregivers	•Identify and treat the reversible medical causes of delirium through a comprehensive initial assessment ^m^ •Non-pharmacological interventions specific to adult cancer patients do not have enough evidence base for a recommendation •Consider deprescribing medication and cancer therapy •Use pharmacological interventions ^n^
Dans et al.	NCCN Guidelines Palliative Care, Version 2.2017	•Risk factors include patient with cancer and moderate to severe pain, nausea, anxiety, depression, shortness of breath, drowsiness, well-being, loss of appetite, and tiredness in the last weeks of life	•Follow different intervention course ^o^ based on length of estimated life expectancy to reduce patient distress
Devlin et al.	Clinical Practice Guidelines for the Prevention and Management of Pain, Agitation/Sedation, Delirium, Immobility, and Sleep Disruption in Adult Patients in the ICU	•Risk factors include benzodiazepine use, blood transfusions, greater age, dementia, prior coma, pre-ICU emergency surgery or trauma, and increasing APACHE and ASA scores •Use validated delirium predictive models ^p^ •Assess ICU patients regularly for delirium with a valid tool like the CAM-ICU or the ICDSC, however the level of arousal may influence the results	•Non-pharmacological bright light therapy is not recommended •Use multicomponent, non-pharmacological interventions ^q^ •Some pharmacologic interventions ^r^ should not be used to prevent delirium •Subsyndromal delirium should not be treated using haloperidol or an atypical antipsychotic •Use a pharmacological treatment like dexmedetomidine for agitated patients during extubation •Pharmacological interventions for delirium using haloperidol, an atypical antipsychotic, or a statin are not recommended
ACR expert panel	ACR Appropriateness Criteria Acute Mental Status Change, Delirium, and New Onset Psychosis	•Risk factors include medical and environmental factors ^s^ •Diagnose delirium using the DSM-V criteria •Use screening tools like the CAM and briefer variants (CAM-ICU, B-CAM)	•Use CT head without contrast for initial imaging of new onset delirium •Consider MRI and contrast enhanced MRI for further evaluation of a brain abnormality (space occupying legion or infection) linked to delirium and previously detected using CT
Fraser Health	Delirium/Restlessness Symptom Guidelines	•Risk factors are usually multi-factorial ^u^ •Assess delirium using the acronym OPQRSTUV ^t^ in addition to laboratory studies ^v^ •Diagnose delirium using the DSM-IV criteria	•Use non-pharmacological interventions ^w^ •Use pharmacological interventions ^x^ based on level of confusion after treating reversible medical causes •Consider palliative sedation when all other measures have failed
Grover and Avasthi	Clinical Practice Guidelines for Management of Delirium in Elderly	•“Robust” risk factors include higher age, presence of cognitive impairment, severe concomitant medical illness and receiving medications however others ^y^ can also contribute to delirium •Diagnose delirium using the DSM-V criteria •Use various screening and diagnosis instruments ^z^ to diagnose the presence and severity of delirium	•Identify and treat reversible medical causes of delirium ^aa^ •Monitor for changes or recurrence in hospital ^ab^ •Use non-pharmacological interventions for support and orientation, maintaining of patient competence, and creating an unambiguous environment ^ac^ •Use pharmacological interventions ^ad^ when non-pharmacological interventions have failed, or when severe agitation is present •Use scales like the Anticholinergic Burden Classification to minimize anticholinergic load •Follow-up after discharge from hospital and educate family on signs of recurrence
Harle et al.	Cancer Care Ontario's Symptom Management Guide-to-Practice: Delirium	•Risk factors are usually multi-factorial, use the acronym DELIRIUM ^ae^ to facilitate assessment •Assess delirium using the acronym OPQRSTUV (adapted from Fraser Health) •Diagnose delirium using the DSM-IV criteria •Screen for delirium using the Mini-Mental State Exam, Confusion Rating Scale, Nursing Delirium Screening Scale, Memorial Delirium Assessment Scale	•Use non-pharmacological interventions and pharmacological interventions based on level of confusion after treating reversible medical causes (adapted from Fraser Health) ^af^
Hogan and McCabe	National Guidelines for Seniors' Mental Health: The Assessment and Treatment of Delirium	•Risk factors are multifactorial ^ag^ •Diagnose delirium using the DSM-IV criteria •Detect and routinely screen for delirium using validated tools ^ah^	•Monitor delirium using reliable tools ^ah^ •Treat reversible medical causes first such as infection, pain, and sensory deficits •Use non-pharmacological interventions to treat and prevent delirium ^ai^ •Remove medication that precipitates of aggravates delirium ^aj^ •Use pharmacological management only if patient is distressed ^ak^ •Only use physical restraints in exceptional circumstances where the benefits outweigh the risks to patient
Jacobi et al.	Clinical Practice Guidelines for the Sustained Use of Sedatives and Analgesics in the Critically Ill Adult	•Diagnose delirium using the DSM-IV criteria •Routinely screen for delirium using the validated CAM-ICU tool	•Use haloperidol as a pharmacological intervention, and monitor for electrocardiographic changes after prescribing
Martin et al.	Evidence and Consensus-based German Guidelines for the Management of Analgesia, Sedation and Delirium in Intensive Care - Short Version	•Screen for delirium using validated tools like the CAM-ICU and the ICDSC	•Use non-pharmacological interventions for example maintaining day-night rhythm, environment reorganisation, cognitive stimulation, and early mobilization •Use antipsychotics for the treatment and prevention of delirium •During substance withdrawal, use alpha-2 agonists and benzodiazepines as a pharmacological intervention •Taper analgesics and sedatives to reduce risk of withdrawal
McNeill et al.	Delirium, Dementia, and Depression in Older Adults: Assessment and Care, Second Edition	•Assess risk factors ^al^ on admission and if a change in condition occurs •Use clinical assessment and validated tools ^am^ to assess patients at risk for delirium at least daily where appropriate or when changes in cognition are observed •Educate family to recognise signs of delirium	•Use multicomponent non-pharmacological ^al^ and pharmacological interventions tailored to risk factors in collaboration with the patient, the family, and the interprofessional team •Use physical restraints as a last resort
Michaud et al.	Delirium: Guidelines for General Hospitals	•Risk factors ^an^ are multifactorial and include predisposing factors on admission, precipitating factors during stay and aggravating environmental factors •Use validated tools ^ao^ for screening, diagnosing, and rating the severity of delirium •The use of electroencephalogram, brain imaging and lumbar puncture is controversial	•Use non-pharmacological interventions ^ap^ •Minimize drug effects and withdrawal symptoms •Use pharmacological interventions ^aq^ when non-pharmacological interventions fail and patient remains agitated •Restraint may be needed for dangerous patients, however a restraint protocol must be used and routinely evaluated
Neufeld et al.	Antipsychotics for the Prevention and Treatment of Delirium	NS	•There is little to no evidence to determine the effect of antipsychotics for prevention of delirium •Second-generation antipsychotics may lower the occurrence of delirium in post-surgical patients •Treating or preventing delirium using haloperidol or second-generation antipsychotics may lead to heart-related side effects and little or no difference in sedation
NICE rapid guideline development group	COVID-19 Rapid Guideline: Managing Symptoms (including at the end of life) in the Community	NS	•Treat reversible medical causes of delirium •Use non-pharmacological interventions like adequate lighting, effective communication, and orientation techniques •Consider a benzodiazepine like levomepromazine based on patient swallowing capacity or level of distress ^ar^
Potter et al.	The Prevention, Diagnosis and Management of Delirium in Older People: Concise Guidelines	•All healthcare professionals can diagnose delirium using the CAM screening tool •Routinely screen all older patients admitted to hospital •Senior doctors and nurses should ensure that doctors in training and nurses are able to recognise and treat delirium	•Treat reversible medical causes first •Incorporate non-pharmacological interventions ^as^ into the care plan of patients at high risk •Minimize the use of sedatives and major tranquilisers •Use one drug only (haloperidol is currently recommended) starting at the lowest dose and increasing every two hours if necessary • Review all medications at least every 24 hours and provide one-to-one care when using pharmacological interventions
Thomson et al.	Diagnosis and Management of Delirium	•Risk factors include advancing age, dementia, hip fracture, previous history of delirium, multiple co-morbidities, and polypharmacy •Screen using validated tool like the 4AT, NEWS2, CAM or CAM-ICU	•Treat reversible medical causes identified by the PINCH-ME ^at^ acronym •Attempt non-pharmacological interventions ^au^ before pharmacological interventions •Use pharmacological interventions ^av^ only if patient is at risk of harming themselves and others, or have very distressing symptoms such as hallucinations •Prevent recurrence by continuing non-pharmacological interventions
Tropea et al.	Clinical Practice Guidelines for the Management of Delirium in Older People	•Diagnose delirium using the DSM-IV criteria •Screen for delirium in all older people using a structured process which includes formative cognitive function assessment and validated tools like the CAM, DRS, and CAM-ICU	•Treat reversible medical causes such as pain, constipation, urinary retention, and hypoxia •Use non-pharmacological interventions ^aw^ to prevent delirium across all health-care settings •Consider pharmacological interventions ^ax^ and review dosage and symptoms continually if severe behavioural or emotional disturbance is present •Include professional follow-up, monitoring, and treatment in the discharge process
Trzepacz et al.	Practice Guideline for the Treatment of Patients with Delirium	•Risk factors are multifactorial ^ay^ •Diagnose delirium using the DSM-IV criteria •Use formal measures for test for delirium ^az^	•Treat reversible medical causes ^ba^ and co-morbid psychiatric disorders first •Psychiatrists should be actively involved in caring and decision processes •Use non-pharmacological interventions to ensure environmental orientation •Use pharmacological interventions ^bb^, preferably a short acting agent and not a benzodiazepine
Virani et al.	Caregiving Strategies for Older Adults with Delirium, Dementia and Depression	•Maintain high attention for prevention, early recognition, and urgent treatment of delirium •Diagnose delirium using the DSM-IV criteria and validated screening methods, and document if delirium is hypoactive or hyperactive	•Implement multi-component interventions (consultation to specialised services, addressing reversible medical causes, using pharmacological interventions, using non-pharmacological interventions like family communication and education) •Monitor interventions on an ongoing basis to address fluctuating course of delirium
Weldon et al.	Guidelines for the Prevention, Recognition and Management of Delirium in Adults in the Acute Hospital Setting	•Risk factors include age of 65 years or older, current hip fracture, cognitive impairment or dementia, and other medical illnesses ^bc^ •Use the acronym DELIRIUM ^bd^ to recognise medical factors •Use the Single Question in Delirium (‘Has [named person]… been more confused in the last 72 hours?’) and CAM as screening tools •Assess for rapid onset of altered cognitive function, inattention, and altered consciousness with a fluctuated course	•Treat delirium as a medical emergency •Treat reversible medical causes first •Use non-pharmacological interventions ^be^ •Consider short term (a week or less) pharmacological intervention using one drug ^bf^ when essential treatment is needed, patient is a risk to themselves or others, or patient is highly agitated or hallucinating
White et al.	Guidelines for the Peri-operative Care of People with Dementia	•Risk factors for post-surgical delirium are multifactorial ^bg^ •Diagnose delirium using formal assessment tools like the CAM and 4AT, paying attention to both hypoactive and hyperactive delirium •Assess delirium using new occurrence or changes in cognition, concentration, perception, behaviour, or physical function	•Treat reversible medical causes ^bg^ •Optimize non-pharmacological interventions such as sleep, nutrition, hydration, sensory aids, bowel, and bladder care •Use single, lowest dose, and shortest use pharmacological intervention (intravenous incremental doses of 0.5 mg haloperidol, or benzodiazepines for alcohol or Parkinsonian-related symptoms)
Young et al.	Delirium: Prevention, Diagnosis and Management	•Risk factors include age of 65 years or older, cognitive impairment or dementia, current hip fracture, and severe illness •Diagnose delirium using the DSM-V criteria •Assess for recent changes or fluctuations in behavior indicative of hyperactive or hypoactive delirium ^bh^ using the validated CAM or CAM-ICU	•Treat reversible medical causes ^bi^ •Use non-pharmacological interventions to prevent delirium ^bj^ •Observe daily for changes •Consider short-term (a week or less) pharmacological intervention using haloperidol if distress is significant or patients are a risk to themselves or others

*Abbreviations*: *ACR* American College of Radiotherapy, *APACHE* Acute Physiology and Chronic Health Evaluation, *ASA* American Society of Anesthesiologists, *BC* British Colombia, *B-CAM* Brief-Confusion Assessment Method, *CAM* Confusion Assessment Method, *CAM-ICU* Confusion Assessment Method-Intensive Care Unit, *COVID-19* Coronavirus disease of 2019, *CT* Computed Tomography, *DRS* Delirium Rating Scale, *DSI* Delirium Symptom Interview, *DSM* Diagnostic and Statistical Manual of Mental Disorders, *ESMO* European Society of Medical Oncology, *ICD* International Classification of Diseases, *ICDSC* Intensive Care Delirium Screening Checklist, *ICU* Intensive care unit, *MRI* Magnetic resonance imaging, *NCCN* National Comprehensive Cancer Network, *NEWS2* National Early Warning Score 2, *NICE* National Institute for Clinical Excellence, *NS* Not stated, *4AT* The 4 ‘A’s Test

Recommended delirium assessment tools included the Confusion Assessment Method (CAM) [24], Confusion Assessment Method-Intensive Care Unit (CAM-ICU) [25], the 4 “A”s Test (4AT) [26, 27], and the Intensive Care Delirium Screening Checklist (ICDSC) [28]. Approximately half (*n* = 14; 52%) of included guidelines explicitly recommended using validated tools for delirium assessment. The most common diagnostic criteria for delirium was the fourth or fifth edition of the Diagnostic and Statistical Manual of Mental Disorders (DSM-IV or DSM-5), mentioned in 11 guidelines (41%). General recommendations for the recognition of delirium included training for healthcare professionals to increase awareness of delirium and delirium treatment options [29–31]. One guideline recommended referring patients with delirium to a trained mental health professional for further evaluation [32].

Recommendations for delirium prevention included pharmacological and non-pharmacological interventions once reversible medical causes of delirium (e.g., infection, pain) were addressed. Non-pharmacological interventions promoted patient orientation (i.e., using clocks, using a calendar, avoiding unnecessary room changes); social contact (i.e., friend and family visits); and comfort (i.e., avoiding unnecessary catheterization, monitoring nutrition and hydration, ensuring working hearing and visual aids) [23]. Adequate analgesia was recommended using a non-opioid medication first, and if an opioid was needed the minimum effective dose was recommended [29] with an opioid rotation in place [30]. Physical restraint was only recommended in exceptional circumstances, when a patient was a risk to themselves or others [33], and a restraint protocol should be used and routinely re-evaluated [34]. Pharmacological management for delirium most commonly included treatment with a benzodiazepine, a first, second, or third-generation antipsychotic [35], or a cholinergic drug [36]. Cancer therapy and medications exacerbating delirium (e.g., benzodiazepines, phenytoin) [33, 35] were recommended to be deprescribed. One guideline provided recommendations for imaging new onset (i.e., incident) delirium using a computed tomography (CT) head scan without contrast. Further evaluation of delirium with suspected brain abnormalities can be performed with contrast enhanced magnetic resonance imaging [37].

### Summary of recommendations for anxiety, depression, and PTSD in delirium

A synthesis of recommendations for guidelines that report on anxiety, depression and PTSD in delirium is presented in Table 4. Two out of the seven guidelines (29%) [23, 38] provided recommendations specific to older adults > 65 years of age.

**Table 4:** Synthesis of recommendations for guidelines reporting on anxiety, depression, and post-traumatic stress disorder in delirium

Author	Guideline name	Anxiety	Depression	Post-traumatic stress disorder
Andersen et al.	Screening, Assessment, and Care of Anxiety and Depressive Symptoms in Adults with Cancer: An American Society of Clinical Oncology Guideline Adaptation	•Treat reversible medical causes of symptoms •Assess and screen based on specific risk factors using validated tools ^a^ •Recommend treatment pathways ^b^ based on the severity of symptoms •Follow-up and assess compliance on a biweekly or monthly basis until remission		
BC Guidelines and Protocols Advisory Committee	Palliative Care for the Patient with Incurable Cancer or Advanced Disease Part 2: Pain and Symptom Management		•Treat reversible medical causes of symptoms first •Differentiate mental symptoms from normal grieving process •Select an antidepressant for pharmacological intervention with the least amount of drug interactions ^c^	
Jacobi et al.	Clinical Practice Guidelines for the Sustained Use of Sedatives and Analgesics in the Critically Ill Adult	•Use pharmacological sedation for agitation only after treating reversible medical causes and providing analgesia		Refer to anxiety recommendations
McNeill et al.	Delirium, Dementia, and Depression in Older Adults: Assessment and Care, Second Edition		•Assess and screen using validated tools ^d^, risk factors ^e^, and collaboration with family caregivers •Assess patients with suspected or present depression for risk of suicide and further refer for an in-depth assessment by a mental health specialist •Develop an individualized treatment plan using a collaborative approach and consider the effects of co-morbid dementia •Use evidence-based pharmacological antidepressants and non-pharmacological ^f^ interventions, educate patient and family caregivers about depression and management options ^g^ •Monitor depression for changes and document effectiveness of treatment	
NICE rapid guideline development group	COVID-19 Rapid Guideline: Managing Symptoms (including at the end of life) in the Community	•Use non-pharmacological interventions to address medical and environmental causes of anxiety •Use a benzodiazepine ^h^ for pharmacological intervention		
Trzepacz et al.	Practice Guideline for the Treatment of Patients with Delirium	•Prioritize treating delirium first over other comorbid psychiatric disorders •Minimize or do not begin pharmacological treatments (antidepressant or anxiolytic) for comorbid psychiatric conditions until delirium has resolved to limit the risk of aggravation		
Virani et al.	Caregiving Strategies for Older Adults with Delirium, Dementia and Depression		•Maintain a high level of attention for early recognition and treatment of depression •Use standardized tools ^i^ to identify depression and pharmacological risk factors ^j^ •Use non-pharmacological ^k^ and pharmacological ^l^ interventions •Facilitate patient-family communication •Monitor depression for changes based on stage of recovery	

*Abbreviations*: *BC* British Colombia, *COVID-19* Coronavirus disease of 2019, *NICE* National Institute for Clinical Excellence

^a^E.g., Generalized Anxiety Disorder 7-item (GAD-7), Patient Health Questionnaire 9 (PHQ-9) (See guideline Table 2, pg. 1612 for additional detail)

^b^E.g., None/mild symptomatology offers supportive care services, moderate symptomatology offers low intensity psychological or pharmacological interventions, severe symptomatology offers high intensity psychological or pharmacological interventions (See guideline Figure 1B, pg. 1610 [depression] and Figure 2B, pg. 1615 [anxiety] for additional detail)

^c^E.g., Selective Serotonin Reuptake Inhibitor, Selective Serotonin Norepinephrine Reuptake Inhibitor, Tricyclic Antidepressant (See guideline Additional file 3: Appendix A, pg. 1-2 for additional detail)

^d^E.g., Geriatric Depression Scale, Patient Health Questionnaire-9 (PHQ-9), Distress Thermometer (See guideline Additional file 3: Appendix H, pg. 141–142 for additional detail)

^e^E.g., Cognitive decline or dementia, social isolation, personal or family history of depression or mood disorder (See guideline Table 4, pg. 71 for additional detail)

^f^E.g., psychotherapy, exercise, electroconvulsive therapy (See guideline Table 5, pg. 76-77 for additional detail)

^g^Key topics include self-management, lifestyle modification, therapeutic interventions, safety, and follow-up care (See guideline Table 6, pg. 78 for additional detail)

^h^E.g., lorazepam, midazolam, haloperidol (See guideline Table 6, pg. 18-19 for additional detail and dosage scheme)

^I^E.g., Diagnostic criteria from the Diagnostic and Statistical Manual (DSM) IV-R, Sig:E Caps, Cornell Scale for depression (See guideline Additional file 3: Appendix K, pg. 141-144 for additional detail)

^j^E.g., Antihypertensives, Antimicrobials, Analgesics (See guideline Additional file 3: Appendix U, pg. 176 for additional detail)

^k^E.g., Education for clients, environment and light therapy, Aromatherapy (See guideline pg. 72 for additional detail)

^l^E.g., Selective serotonin reuptake inhibitors (SSRI), tricyclic antidepressants are not recommended (See guideline pg. 73-74 for additional detail)

Four out of the seven guidelines (57%) provided recommendations specific to the management and prevention of anxiety in delirium. Andersen et al. [32] recommended screening based on validated tools such as the Beck Anxiety Inventory (BAI) [39], Generalized Anxiety Disorder (GAD-7) [40], Hospital Anxiety and Depression Scale (HADS) [41], or Spielberger State-Trait Anxiety Inventory (STAI) [42]. Non-pharmacological interventions were recommended for management of environmental and medical causes of anxiety [43]; however, no specific interventions were recommended. Pharmacological recommendations included the use of a benzodiazepine [43], while minimizing anxiolytics until delirium has resolved to limit delirium aggravation [36]. General treatment considerations included treating reversible medical causes of symptoms first [32], and treating delirium first over other psychiatric disorders [36]. Jacobi et al. indicated that pharmacological sedation should only be used after reversible medical causes were treated and analgesia was provided [20]. Andersen et al. recommended different treatment pathways based on the severity of symptoms of anxiety (e.g., mild symptomatology may require referral to supportive care services while severe symptomatology may require high intensity psychological and pharmacological intervention) [32].

Five out of the seven guidelines (71%) provided recommendations specific to the recognition and management of depression in delirium. Risk factors for depression included patient factors (i.e., cognitive decline or dementia, social isolation, personal or family history of depression or mood disorder) [23] and previous use of certain medications (i.e., antihypertensives, antimicrobials, and analgesics) [38]. Tools recommended for depression screening included the Sig: E. Caps [44], Cornell Scale for Depression [45], and Geriatric Depression Scale [46]. A variety of non-pharmacological interventions for depression were presented and these included: psychotherapy, exercise, electroconvulsive therapy, aromatherapy, and light therapy [23, 38]. All five guidelines mentioned pharmacological interventions and these included antidepressants such as selective serotonin reuptake inhibitors (SSRI) and tricyclic antidepressants; however, selection was recommended to be based on a medication with the fewest drug interactions [47] and with limited duration of therapy during delirium [36] as it may worsen delirium symptoms. General treatment considerations included first treating reversible medical causes, differentiating psychiatric symptoms from normal grieving process for those with cancer or advanced disease, and referring patients with a risk of suicide for an in-depth assessment with a mental health specialist [23]. One guideline recommended that an individualized treatment plan should be developed based on levels of depression severity, and the patient should be monitored for changes in behavior and followed-up every two weeks after intervention [32].

A single guideline out of the seven (14%) provided a recommendation specific to PTSD in delirium, and referred to using pharmacological sedation for agitation only after treating reversible medical causes of symptoms and providing adequate analgesia [20].

## Discussion

In this systematic review, we provide an updated synthesis of CPGs for the diagnosis and management of delirium in patients admitted to an acute care setting. This review also provides a new analysis of CPGs with recommendations for the diagnosis and management of anxiety, depression, or PTSD in adults with delirium in acute care. Recommendations for the diagnosis of psychiatric disorders in delirium included screening with tools and assessment of risk factors, while recommendations for the management of psychiatric disorders in delirium included treating reversible medical causes first, utilizing non-pharmacological and pharmacological interventions, and monitoring the patient for changes in cognition. This review highlights the lack of published CPGs for healthcare professionals to recognize and prevent psychiatric disorders in adults with delirium using evidence-based practice.

Recommendations, if present, were often vague (e.g., recommending pharmacological agents without specifying by type or name of drug, lack of specific diagnosis criteria or flowcharts). This may be due to the lack of studies with a low risk of bias (i.e., randomized controlled trials [RCTs]) being performed on the prevention and treatment of psychiatric disorders in adults with delirium in acute care; or a deficit in updating guidelines to reflect current evidence-based practice. Clinical practice is altered constantly as new information is acquired, and new studies are performed [48]. In this review, 70% of included guidelines were published before 2019, and many have no explicit update information or criteria (AGREE II Domain 3, item 14). Up to date clinical practice guidelines are needed to ensure current evidence-based practice and better patient outcomes. Ideally, guideline developers should strive to improve not only the CPG update procedure and search terms used for evidence acquisition described in the AGREE II Domain 3, Rigour of Development but all AGREE II Domains with the lowest scores: Domain 5, Applicability; and Domain 6, Editorial Independence. Guideline developers should ensure that financial implications of guideline implementation have been considered and that clear auditing criteria are described to satisfy the Applicability Domain. Lastly, for the Editorial Independence Domain, the reporting of guideline developers’ competing interests and explicit funding statements should be included.

Healthcare professionals have reported feeling underprepared when dealing with patients with psychiatric disorders in the ICU [49] and may benefit from CPG recommendations to guide patient care. Some guidelines presented screening tools to facilitate the recognition of psychiatric disorders in delirium. Early identification of psychiatric disorders in delirium using standardized screening tools is important in the implementation of preventative interventions [50], which could contribute to a lowered psychological burden [51]. For the prevention of anxiety, depression, and PTSD in delirium, both pharmacological and non-pharmacological interventions were discussed. Non-pharmacological interventions (i.e., music therapy, mind-body interventions by family members or healthcare professionals, counselling) have been previously used in the ICU to reduce ICU-related distress without the need for sedative drugs [52]. Pharmacological interventions for anxiety or depression should be carefully assessed, as antidepressants and benzodiazepines are linked to worsened delirium [53, 54]. In response, guidelines recommended waiting until delirium has resolved to prescribe pharmacological interventions for anxiety or depression [36].

Our work aimed to update a 2017 systematic review on the quality of CPGs for use in the treatment of delirium within acute care [14]. Similarly, to this review, we identified many guidelines not kept up to date, as well as CPGs that achieved low AGREE scores in the domains of Applicability and Editorial Independence. However, our review reveals newfound differences in guideline target users as well as patient population, such that CPGs identified in our review were targeted to groups other than healthcare professionals (e.g., family caregivers, health system leaders, and policy makers). In addition, we found a higher proportion of CPGs that included recommendations for the general hospitalized population. This may represent the increased knowledge and recognition of delirium among members of the clinical care team (apart from healthcare professionals) in the acute care setting over the last 10 years.

The information provided in this systematic review must be taken in the context of relevant limitations. Most guidelines included in this review originated from Canada, the United States of America, or the United Kingdom, and were written primarily in English. Approaches to delirium recognition and treatment, in addition to the use of protocols for the management of delirium, are known to vary across countries [55]; guidelines included in our study may under-represent perspectives from other countries. While most clinicians understand CPGs to be helpful tools, their use in practice may be limited by the inflexibility of certain recommendations in specialized or unusual cases [56]. As well, to ensure reliability, two authors completed study screening, data extraction, and guideline quality assessment independently and in duplicate. Considering that reliability statistics in systematic reviews are sensitive to 'true prevalence' in the data—if the true prevalence of a population is high or low, agreement expected by chance increases and the magnitude of kappa goes down—reliability statistics were not conducted. Additionally, guidelines that were excluded from this review in the full-text stage due to lack of endorsement or availability of full-text may have provided additional recommendations that were missed.

This synthesis of guideline recommendations for delirium and for anxiety, depression, and PTSD in delirium provides a succinct guide for healthcare professionals. Based on the existing literature, current recommendations for the diagnosis and management of delirium and of anxiety, depression, and PTSD in delirium are minimal, but may help inform patient care. More recommendations for the diagnosis and management of psychiatric disorders in delirium are needed, and the conduct of RCTs for interventions of interest could facilitate higher quality evidence-based recommendations.

## Conclusion

The evidence base is minimal for clinical practice guidelines that report on the diagnosis and management of symptoms of possible psychiatric disorders in adult patients with delirium in the acute care setting. Patients with delirium that display symptoms of psychiatric disorders may require specific evaluation during their hospitalization to ensure that psychiatric disorders are identified, and management plans are developed for this patient population in the follow-up period.

### Supplementary Information

**Additional file 1:** **Appendix 1.** PRISMA 2020 checklist. Table containing the PRISMA 2020 checklist.

**Additional file 2:** **Appendix 2.** Database and grey literature search strategies. List of all search strategies used for MEDLINE, EMBASE, PsycINFO, and CINAHL databases, and a grey literature search tool.

**Additional file 3:** **Appendix 3.** Footnotes of Table 3: Synthesis of recommendations for delirium. Contains footnotes of Table 3.

## Data Availability

Data sharing is not applicable to this article as no datasets were generated or analyzed during the current study.

## Abbreviations

AGREE: Appraisal of Guidelines for Research & Evaluation Instrument
BAI: Beck Anxiety Inventory
CADTH: Canadian Agency for Drugs and Technologies in Health
CAM: Confusion Assessment Method
CAM-ICU: Confusion Assessment Method Intensive Care Unit
CPG: Clinical Practice Guideline
CT: Computed tomography
GAD: General anxiety disorder
GRADE: Grading of Recommendations, Assessment, Development and Evaluations
HADS: Hospital Anxiety and Depression Scale
ICDSC: Intensive Care Delirium Screening Checklist
ICU: Intensive care unit
PRISMA: Preferred Reporting Items for Systematic Review and Meta-Analyses
PTSD: Post traumatic stress disorder
RCT: Randomized controlled trial
STAI: State-Trait Anxiety Inventory
