# Supplementary material for: Clinical practice guideline recommendations for diagnosis and management of anxiety and depression in hospitalized adults with delirium: a systematic review
Source: Syst Rev. 2023 Sep 25;12:174. doi: 10.1186/s13643-023-02339-6 (PMC10519074; doi:10.1186/s13643-023-02339-6)
Supplement: Supplementary file 2 — Additional file 2: Appendix 2. Database and grey literature search strategies. List of all search strategies used for MEDLINE, EMBASE, PsycINFO, and CINAHL databases, and a grey literature search tool. [file 13643_2023_2339_MOESM2_ESM.docx]

**Appendix 2.** Database and grey literature search strategies

*MEDLINE*

1. exp Confusion/

2. confusion*.tw.

3. deliri*.mp.

4. ((ICU or intensive care unit or post-operative) and psychos*).mp.

5. or/1-4

6. exp clinical pathway/

7. exp clinical protocol/

8. exp consensus/

9. exp consensus development conference/

10. exp consensus development conferences as topic/

11. critical pathways/

12. exp guideline/

13. guidelines as topic/

14. exp practice guideline/

15. practice guidelines as topic/

16. health planning guidelines/

17. guideline adherence/

18. (guideline or practice guideline or consensus development conference or consensus development conference, NIH).pt.

19. (position statement* or policy statement* or practice parameter* or best practice*).ti,ab,kf,kw.

20. (standards or guideline or guidelines).ti,kf,kw.

21. ((practice or treatment* or clinical) adj guideline*).ab.

22. (CPG or CPGs).ti.

23. consensus*.ti,kf,kw.

24. consensus*.ab. /freq=2

25. ((critical or clinical or practice) adj2 (path or paths or pathway or pathways or protocol*)).ti,ab,kf,kw.

26. recommendat*.ti,kf,kw.

27. (care adj2 (standard or path or paths or pathway or pathways or map or maps or plan or plans)).ti,ab,kf,kw.

28. (algorithm* adj2 (screening or examination or test or tested or testing or assessment* or diagnosis or diagnoses or diagnosed or diagnosing)).ti,ab,kf,kw.

29. (algorithm* adj2 (pharmacotherap* or chemotherap* or chemotreatment* or therap* or treatment* or intervention*)).ti,ab,kf,kw.

30. or/6-29

31. 5 and 30

*EMBASE*

1. Exp Confusion/

2. Exp Delirium/

3. Exp Intensive Care Psychosis/

4. confusion*.tw.

5. deliri*.mp.

6. ((ICU or “intensive care unit” or post-operative) and psychos*).mp.

7. or/1-6

8. exp clinical pathway/

9. exp clinical protocol/

10. exp consensus/

11. exp consensus development conference/

12. exp consensus development conferences as topic/

13. critical pathways/

14. guidelines/

15. guidelines as topic/

16. exp practice guideline/

17. practice guidelines as topic/

18. health planning guidelines/

19. guideline adherence/

20. (guideline or practice guideline or consensus development conference or consensus development conference, NIH).mp.

21. (position statement* or policy statement* or practice parameter* or best practice*).ti,ab,kw.

22. (standards or guideline or guidelines).ti,kw.

23. ((practice or treatment* or clinical) adj guideline*).ab.

24. (CPG or CPGs).ti.

25. consensus*.ti,kw.

26. consensus*.ab. /freq=2

27. ((critical or clinical or practice) adj2 (path or paths or pathway or pathways or protocol*)).ti,ab,kw.

28. recommendat*.ti,kw.

29. (care adj2 (standard or path or paths or pathway or pathways or map or maps or plan or plans)).ti,ab,kw.

30. (algorithm* adj2 (screening or examination or test or tested or testing or assessment* or diagnosis or diagnoses or diagnosed or diagnosing)).ti,ab,kw.

31. (algorithm* adj2 (pharmacotherap* or chemotherap* or chemotreatment* or therap* or treatment* or intervention*)).ti,ab,kw.

32. or/8-31

33. 7 and 32

*PsycINFO*

1. Exp Mental Confusion/

2. Exp Delirium/

3. confusion*.tw.

4. deliri*.mp.

5. ((ICU or “intensive care unit” or post-operative) and psychos*).mp.

6. or/1-5

7. exp Evidence Based Practice or exp Clinical Practice/

8. exp Clinical Practice/ or exp Treatment Effectiveness Evaluation or exp Treatment Guidelines/

9. exp Group Decision Making/ or exp Decision Making/ or exp Algorithms/ or Expert Systems/

10. exp Rating Scales/ or exp Evaluation/ or exp Psychometrics/

11. exp Diagnosis/ or exp Disease Management/

12. exp Treatment Planning/

13. exp Treatment Compliance/ or exp Health Personnel/

14. (guideline or practice guideline or consensus development conference or consensus development conference, NIH).mp.

15. (position statement* or policy statement* or practice parameter* or best practice*).ti,ab.

16. (standards or guideline or guidelines).ti.

17. ((practice or treatment* or clinical) adj guideline*).ab.

18. (CPG or CPGs).ti.

19. consensus*.ti.

20. consensus*.ab. /freq=2

21. ((critical or clinical or practice) adj2 (path or paths or pathway or pathways or protocol*)).ti,ab.

22. recommendat*.ti.

23. (care adj2 (standard or path or paths or pathway or pathways or map or maps or plan or plans)).ti,ab.

24. (algorithm* adj2 (screening or examination or test or tested or testing or assessment* or diagnosis or diagnoses or diagnosed or diagnosing)).ti,ab.

25. (algorithm* adj2 (pharmacotherap* or chemotherap* or chemotreatment* or therap* or treatment* or intervention*)).ti,ab.

26. or/7-25

27. 6 and 26

*CINAHL*

1. TX Acute Confusion

2. (MH Confusion)

3. (MH ICU Psychosis)

4. TX confusion*

5. TX deliri*

6. TX “intensive care unit psycho*”

7. TX “post-operative psychos*”

8. S1 OR S2 OR S3 OR S4 OR S5 OR S6 OR S7

9. TI guideline* OR TI guidance OR TI recommendation* OR TI consensus* OR TI best practice* OR TI statement* OR TI standard* OR TI practice parameter* OR TI position paper OR TI position stand

10. (MH "Practice Guidelines") OR (MH "Consensus" ) OR (PT practice guidelines)

11. S9 OR S10

12. S8 AND S11

*Alternate:*

*(TX Acute Confusion or (MH Confusion) or (MH ICU Psychosis) or TX confusion* or TC deilri* or TX "intensive care unit psycho*" or TX "post-operative psychos*")*

*AND*

*(TI guideline* OR TI guidance OR TI recommendation* OR TI consensus* OR TI best practice* OR TI statement* OR TI standard* OR TI practice parameter* OR TI position paper OR TI position stand) OR ((MH "Practice Guidelines") OR (MH "Consensus") OR (PT practice guidelines))*

*GREY LITERATURE*

All websites (n=21) listed in the Canadian Agency for Drugs and Technologies in Health (CADTH) Grey Matters Lite tool available from <https://www.cadth.ca/resources/finding-evidence/grey-matters>. This tool was developed specifically for finding and reviewing CPGs.
