# Supplementary material for: Clinical practice guideline recommendations for diagnosis and management of anxiety and depression in hospitalized adults with delirium: a systematic review
Source: Syst Rev. 2023 Sep 25;12:174. doi: 10.1186/s13643-023-02339-6 (PMC10519074; doi:10.1186/s13643-023-02339-6)
Supplement: Supplementary file 3 — Additional file 3: Appendix 3. Footnotes of Table 3: Synthesis of recommendations for delirium. Contains footnotes of Table 3. [file 13643_2023_2339_MOESM3_ESM.docx]

**Appendix 3.** Footnotes of Table 3: Synthesis of recommendations for delirium.

^a^ E.g., advanced age, pain, cognitive impairment (See guideline Table 3.1, pg. 17 for additional detail)

^b^ E.g., palliative care philosophy and principles, incidence of delirium at end of life, precipitating and predisposing factors (See guideline Table 7.1, pg. 37-38 for additional detail)

^c^ E.g., Confusion Assessment Method (CAM), CAM-Intensive care unit (CAM-ICU), Delirium Rating Scale-Revised 98 (DRS-R 98) (See guideline Appendix E, pg. 52-62 for additional detail)

^d^ E.g., fall prevention protocol, clear communication, sensory aids, music therapy (See guideline pg. 27-29 for additional detail)

^e^ E.g., cognition, vital signs, sleep patterns (See guideline Table 4.4, pg. 26 for additional detail)

^f^ E.g., benzodiazepines, meperidine, oxybutynin (See guideline Table 6.1, pg. 36 for additional detail)

^g^ Avoid sedatives and use haloperidol to control hallucinations (See guideline Appendix A, pg.1 for dosing scheme)

^h^ Avoid benzodiazepines and start with least sedating antipsychotic (See guideline Appendix A, pg.1 for dosing scheme)

^I^ Sedate with benzodiazepine in addition to antipsychotic treatment and palliative care consult (See guideline Appendix A, pg.1 for dosing scheme)

^j^ E.g., Mini-Mental State Examination, Delirium Rating Scale-Revised 98, Delirium Symptom Interview (See guideline pg. 44-45 for additional detail)

^k^ E.g., primary or secondary central nervous system tumor, encephalopathy from radiation to brain, chemotherapy (See guideline Table 1, pg. iv145 for additional detail)

^l^ E.g., physical complications like glucose abnormalities, medications like anxiolytics, other status or comorbidities like hearing impairment (See guideline Table 1, pg. iv145 for additional detail)

^m^ E.g., history, medication review, laboratory tests, physical examination (See guideline Figure 2, pg. iv150 for additional detail)

^n^ E.g., haloperidol, olanzapine, aripiprazole (See Table 4, pg. iv157-iv158 for additional detail)

^o^ E.g., years to months: assess prognostic awareness, facilitate advance care planning (See guideline PAL-27, pg. 991 for additional detail), months to weeks or weeks to days: refer to hospice care agencies, provide education, refer to grief counseling (See guideline PAL-28, pg. 992 for additional detail

^p^ E.g., PREdiction of DELIRium in ICu patients (PRE-DELIRIC) model, Early (E)-PRE-DELIRIC model (See guideline pg. e843 for additional detail)

^q^ E.g., reorientation, cognitive stimulation, use of clocks, minimizing light or noise (See guideline pg. e847-e848 for additional detail)

^r^ E.g., haloperidol, atypical antipsychotic, dexmedetomidine (See guideline pg. e845-e846 for additional detail)

^s^ E.g., sepsis or infection, hypoxia, hyperglycemia, sedatives, sleep deprivation (See guideline pg. S33 for additional detail)

^t^ E.g., Onset, Provoking/Palliating, Quality, Region/Radiation, Severity, Treatment, Understanding, Values (See guideline Table 1, pg.3 for additional detail)

^u^ E.g., neoplastic, infection/inflammatory, metabolic, drug effects (See guideline pg.6-8 for additional detail)

^v^ E.g., serum electrolytes, urea, creatinine, glucose (See guideline pg. 5 for additional detail)

^w^ E.g., calm and quiet environment, night light, prevent overstimulation by keeping visitors to a minimum (See guideline pg. 9 for additional detail)

^x^ E.g., restless and confused but cooperative: haloperidol, methotrimeprazine; delirium with paranoia, confusion and/or aggression: haloperidol or methotrimeprazine or chlorpromazine (See guideline pg. 10 for additional detail and dosing scheme)

^y^ E.g., socio-demographic variables, baseline medical status, past history (See guideline Table 2 for additional detail)

^z^ E.g., Delirium Observation Screening Scale/Delirium Observation Scale, Global Attentiveness Rating, Intensive care delirium screening checklist (See guideline Table 3 for additional detail)

^aa^ E.g., functioning of bladder and bowel, infection, poisoning (See guideline Table 4, 5, 6 for additional detail)

^ab^ E.g., rate severity of symptoms over time, seral assessment of cognitive functions, review sleep chart (See guideline Table 8 for additional detail)

^ac^ E.g., providing support and orientation: using simple language, slow-paced speech, reorientation; unambiguous environment: remove harmful and unfamiliar object, avoid sensory deprivation; maintaining competence: recognise and correct sensory impairments, early mobilization (See guideline Table 9 for additional detail)

^ad^ E.g., antipsychotics such as haloperidol, benzodiazepine such as lorazepam, cholinesterase inhibitors such as donepezil (See guideline Table 10 for additional detail)

^ae^ E.g., D: drugs, dehydration, depression; E: electrolyte, endocrine dysfunction; L: liver failure; I: infection; R: respiratory problems, retention of waste; I: increased intracranial pressure; U: uremia, undertreated pain; M: metabolic disease, metastasis to brain (See guideline Figure 1 and Table 3, pg. 5-6 for additional detail)

^af^ E.g., non-pharmacological: encourage family to be present in a calming way, provide calm, quiet environment (See guideline pg. 6-7 for additional detail); pharmacological: haloperidol, risperidone, olanzapine (see guideline pg. 7-9 and Table 4, pg. 10-12 for additional detail)

^ag^ E.g., socio-demographic, physical status, mental status (See guideline Table 2.1, pg. 26 and Table 3.1, pg. 31 for additional detail)

^ah^ E.g., Confusion Assessment Method, Mini-Mental Status Examination, Montreal Cognitive Assessment (See guideline pg. 29 for additional detail)

^ai^ E.g., mobility and function, safety, communication, behavioural management, environment (See guideline Table 2.2, pg. 27 and Table 3.3, pg. 33 and Table 4.3, pg. 40 and pg. 35-38 for additional detail)

^aj^ E.g., sedative-hypnotics, narcotics, anticholinergic drugs (See guideline Table 4.1, pg. 39 for additional detail)

^ak^ E.g., typical antipsychotics, atypical antipsychotics, benzodiazepines (See guideline pg. 41-16 for additional detail)

^al^ E.g., cognitive impairment treated with cognitive orientation, isolation treated with family visits, sensory impairment treated with aids (See guideline Appendix G, pg. 133-134 for additional detail)

^am^ E.g., 4AT, Confusion Assessment Method Instrument, Delirium Observation Scale (See guideline Appendix H, pg. 136-138 for additional detail)

^an^ E.g., central nervous system factors, metabolic factors, systemic medical factors (See guideline Table 2, pg. 374 for additional detail)

^ao^ E.g., screening: Mini-Mental State Exam, Confusion Assessment Method/Blessed Orientation-Memory-Concentration; diagnosis: Confusion Assessment Method; Severity: Delirium Rating Scale or Memorial Delirium Assessment Scale (See guideline Table 6, pg. 377 for additional detail)

^ap^ E.g., sleep promotion, adequate hydration, clocks and calendars (See guideline Table 3, pg. 375 for additional detail)

^aq^ E.g., haloperidol, risperidone, quetiapine (See guideline Table 7, pg. 377 for additional detail)

^ar^ E.g., haloperidol 0.5 mg to 1 mg at night and every two hours when required, levomepromazine 12.5mg to 25 mg subcutaneously as starting dose (See guideline Table 6, pg. 18-19 for additional detail and dosing scheme)

^as^ E.g., appropriate lighting for time of day, use of clocks and calendars, good diet (See guideline pg. 304-305 for additional detail)

^at^ E.g., P: pain, I: infection/intoxication, N: nutrition, C: Constipation, H: hydration/hypoxia, M: medication, E: environmental

^au^ E.g., reduce noise, encourage mobility, avoid relocation to different ward (See guideline pg. 10-11 for additional detail)

^av^ E.g., haloperidol, risperidone, lorazepam (See guideline pg. 13 for additional detail and dosing scheme)

^aw^ E.g., appropriate lighting for time of day, quiet environment especially at rest times, clearly visible clock and calendar (See guideline Table 2, pg. 152 for additional detail)

^ax^ E.g., haloperidol, olanzapine, risperidone (See guideline Figure 2, pg. 154 for additional detail and dosing scheme)

^ay^ E.g., central nervous system disorder, systemic illness, withdrawal (See Table 1, pg. 14 and Table 2, pg. 15 for additional detail)

^az^ E.g., screening instruments: Confusion Rating Scale, Clinical Assessment of Confusion-A; diagnostic instruments: Confusion Assessment Method, Delirium Scale; delirium symptom severity scale: Delirium Rating Scale, Memorial Delirium Assessment Scale; Laboratory tests: electrolytes, electroencephalogram (See guideline Table 3, pg. 18 and pg. 15-16)

^ba^ E.g., hypoglycemia, hypoxia, hyperthermia (See guideline Table 4, pg. 19 for additional detail)

^bb^ E.g., antipsychotics like haloperidol and chlorpromazine, benzodiazepines like lorazepam, cholinergics like physostigmine (See guideline pg. 22-27 for additional detail)

^bc^ E.g., infections, hyperkalemia, stroke, stroke, surgery (See guideline Table 1, pg. 2-3 for additional detail)

^bd^ D: drugs/dehydration, E: electrolytes, L: level of pain, I: infection, R: respiratory function, I: impaction/constipation, U: urine retention, M: metabolic disorder

^be^ E.g., orientate patient frequently, optimize sensory deficits, explain unfamiliar noises (See guideline Table 3, pg. 5-7 for additional detail)

^bf^ E.g., haloperidol, olanzapine, lorazepam (See guideline pg. 8 and Table 4, pg. 7 for additional detail)

^bg^ E.g., drugs, hypoxia, hypotension, pain, infection (See guideline Appendix 2, pg. 372 for additional detail)

^bh^ E.g., worsened concentration, confusion, visual or auditory hallucinations, restlessness, or reduced movement (See guideline pg. 11 for additional detail)

^bi^ E.g., treating infection, optimize oxygen for hypoxia, provide adequate analgesia (See guideline pg. 11-14 for additional detail)

^bj^ E.g., provide appropriate lighting and clear signage, reorienting, introducing cognitively stimulating activities (See guideline pg. 11-14 for additional detail)
